# Substantial Self-Knowledge and the Necessity of Avowal

**DOI:** 10.1007/s10790-022-09916-3

**Published:** 2022-11-29

**Authors:** Naomi Kloosterboer

**Affiliations:** Philosophy Department, Vrije Universiteit Amsterdam, Boelelaan 1105, 1081 HV Amsterdam, The Netherlands

## Introduction

A central intuition regarding self-knowledge is that if I say (or think) that I believe that it is raining – to use a familiar example – I do not merely state a fact about my mental life but also express my view of the world: I take it to be the case that it is raining. The notion of avowal is supposed to capture this duality of perspectives: whilst occupying one’s first-person perspective, one self-attributes a mental attitude, which is a fact that is supposed to be true independent of one’s own perspective. Another way of putting this is that avowal is a self-attribution of an intentional mental attitude that is *first-personal* and *commissive*.^1^ A person can only avow their *own* mental attitudes, not anyone else’s, and thereby *occupies* or *commits* to the perspective inherent in the attitude. In the case of belief, this means that my avowal expresses my ‘own present commitment to the truth of the proposition in question’ (Moran 2001, 86).^2^ Many grant that avowal and its commissive form are essential to the first-person character of self-knowledge – which I call *the commitment view*.

However, only a few argue that avowal remains essential in achieving self-knowledge of one’s *substantial* mental attitudes, i.e., attitudes that are significant to a person’s life and self-conception, such as one’s values, deeper desires, and cares. In fact, current orthodoxy is skeptical that avowal has any role to play in acquiring substantial self-knowledge.^3^ According to this *skeptical view* of avowal, such attitudes are often distorted in the first-person perspective and are better reflected in someone’s behavior, i.e., in the relevant patterns of action and reaction. Rather than avowing an attitude, interpreting these patterns is supposed to provide substantial self-knowledge.

In the literature, two defenses of the commitment view against the skeptical view can be found. It is either claimed that despite the lack of application to substantial attitudes, the connection between avowal and commitment tells us something *fundamental* about self-knowledge.^4^ Or it is claimed that avowal is necessary for substantial self-knowledge, but should be supplemented with other necessary conditions, such as self-regulation.^5^ I explore a new and different response. Where the first response seeks to defend the importance of avowal despite a lack of application to substantial self-knowledge, and where the second response seeks to undermine the starting point of the skeptical view, namely that our avowals *will* resonate in our behavior if we regulate ourselves properly, I am interested in the importance of avowal *even in the face of* the possibility of a lack of alignment between our words and deeds.

The paper proceeds by first clarifying *the commitment view* (section 2) and *the skeptical view* (section 3). I will then offer two arguments against the skeptical view. First, I will show that substantial mental attitudes cannot, as the skeptical view needs to assume, be discovered in patterns of action and reaction (section 4). Secondly, I will inquire the complex relation between avowal and patterns of action and reaction and argue that a gap between the two cannot be determined nor understood without the agent’s perspective, including their avowal (section 5). Both arguments establish that the skeptic cannot deny a necessary role for avowal. This reflects the agential nature of substantial mental attitudes and challenges the dominant skeptical view of substantial self-knowledge.

## The commitment view

A central intuition of the commitment view is that it just seems bizarre to say that I believe the bank is open today, that I desire my coffee black, or that I am curious about a colleague’s project without taking up the perspective of the attitudes. Such self-attributions, if sincere, do not make sense if I not actually take it to be the case that the bank is open today, that coffee tastes best if black, and that the colleague’s project is interesting and worth my attention. The same intuition still seems to hold in case of substantial mental attitudes: if I say that I care about my job, something seems off if I do not view my job as worthwhile. In this section, I will further explicate this intuition, apply it to substantial attitudes, and state how avowal, commitment, and substantial self-knowledge relate in the commitment view.

### Commitment and the perspective of the agent

The intuition of the commitment view, in a more abstract form, is that a person’s mental attitudes aren’t *mere* psychological facts about her but also express her relation to what the attitudes are about, her *stance* or *grasp* to what goes on in her life and around her.^6^ For instance, an intentional mental attitude such as fear that the drought will ruin the communal garden, expresses how one relates to what goes on in one’s life. In this case, the agent’s fear of the drought expresses her commitment to seeing the drought as a danger to the communal garden.^7^ Avowal is thus seen as essential to the first-person character of self-knowledge, because by expressing commitment, it expresses the unique relation an agent has to her own mental life.

The language of commitment is important in this respect: it signifies the agency and normativity that is involved in having and knowing one’s intentional mental attitudes. At the same time, I use the language of commitment to stay away from language such as “reflective endorsement” that is often interpreted as over-intellectualizing or rationalizing our mental lives.^8^ A recent paper by Casey Doyle (2018, 440) broadly captures the idea of commitment that I am after:

At a first pass, the phenomenon in question is that when one takes the first-person perspective on a reason-responsive attitude, one cannot be indifferent to the commitment it embodies. Taking the first-person stance on the attitude seems to involve taking the stance of the attitude on the world, or, as I will put it, it involves consciously occupying the perspective of the attitude known.

Consciously occupying the perspective of being curious about a colleague’s project is to see that project as worthwhile. I need not explicitly judge that there are sufficient reasons to take the project to be worthwhile (what some take “reflective endorsement” to be). Rather, the normativity of commitment here lies in the idea that the agent is in touch with the perspective of the attitude and that the perspective expresses an *orientation* in the space of reasons. Not as judgment about the quality of those reasons, but as positioning oneself (in thought and experience) to what is true, beautiful, valuable, worthwhile, healthy, etcetera.^9^

Seeing *p* as true (an orientation in the space of reasons) should thus be separated from seeing one’s reasons for taking *p* to be true as good or sufficient. However, commitment to the perspective of the attitude comes with normative pressure to view one’s reasons in this way. This is sometimes expressed as the idea that, from the first-person perspective, the question of whether I believe that *p* and whether *p* is true, i.e., whether *to* believe that *p*, are inherently connected.^10^ Importantly, that there is a relation between these questions does not mean that, from the agent’s own point of view, there is no difference between the questions. An agent can, does, and sometimes should distinguish them. Rather, it means that, from the agent’s point of view, the normative question *is given application*. Simply discarding the question makes no sense if one indeed occupies the perspective of believing that *p*.^11^

One implication of the normative pressure that comes with occupying the perspective of an attitude is that, all else equal, the attitude will be part of a relevant pattern of actions and reactions. For instance, in case of belief that *p*, committing oneself to the truth of *p* comes with normative pressure to use *p*, if relevant, as a guiding factor in one’s thought and action. If the perspective of belief consists of being committed to the truth of the proposition believed, one *should*, on pain of irrationality, use it as a premise in reasoning. That one should use the proposition believed as a guiding factor in one’s thought and action is compatible with the fact that one (sometimes) does not or even should not use it as such. However, if one does not use it as a premise, one should, on pain of irrationality, consider it a failure and feel normative pressure to either reconsider one’s belief or one’s reasoning.^12^

Of course, all else is not always equal. What if, as is all too familiar, patterns of action and reaction contain conflicting elements? For instance, how to account for so-called *recalcitrant* or *persistent* attitudes? Consider Casey Doyle’s description of fear of flying:

While far from phobic, I am uneasy on planes. I often feel fear as a plane takes off. I realize that this is not appropriate. I am aware that flying is far safer than many things I do on a daily basis without worry or regret. I do not endorse the fear and wish I did not experience it. One might be inclined to describe such a state as an alien presence in my mind. But while there might be cases like that, recalcitrance is not restricted to them. My own fear makes sense to me to a degree, and the perspective on the flight it represents is one that I occupy. I feel compelled to embrace the idea that flying is dangerous, despite what I know. I occupy this perspective while judging that it is fine to fly. In a word: it is a mess inside my mind, but I have a first-person perspective on this mess. Simply having an eye on the reasons would miss this, because it would exclude competing voices. (Doyle 2019, 441-442)

In this passage, Doyle emphasizes that we *do* consciously occupy the perspective of a recalcitrant attitude and that, therefore, this perspective is different from an epistemic judgment about one’s reasons. But can it still be understood as an orientation in the space of reasons if that orientation involves conflicting positions?

It seems to me that there are numerous situations in which an agent is pulled in different directions. Consider a dear friend who emigrates to another country, about which one feels excited and sad at the same time. Or think about a birthday party invitation, where loyalty and deadlines pull one in different directions. In a similar vein, when I consider the statistics regarding flying, I believe flying is safe, but when imagining or experiencing being on a plane, the direct feeling of being out of control, the height of the plane, and an image of a plane crashing might get a hold on me. The space of reasons seems to consist not only of unified all-things-considered judgments but of all experiences and considerations relevant to orient oneself to the truth, beautiful, valuable, worthwhile, healthy, etcetera.

In fact, if the recalcitrant fear were not an orientation in the space of reasons, including its normative ramifications, why would it even conflict with the belief that flying is safe? It is precisely because fear is an orientation towards what is true and what is to be done (e.g., avoid danger), that the fear contrasts with the belief. Given the conflict with my all-things-considered judgment about the safety of flying, I actively take care not to let “the perspective of fear” and those features of what goes on in my life to which my fear is attached, get too much of a hold on me and, for instance, figure in my practical reasoning.^13^

In sum, I fully agree that conflict in an agent’s mental life, the “mess” inside her mind, is a normal feature of the first-person perspective. However, I think this is compatible with and can only be conceptualized if consciously occupying the perspective of the attitude is understood as an orientation in the space of reasons. Hence, what this section has clarified is that the language of commitment depicts the idea that, from the first-person perspective, having a mental attitude is inherently connected to being normatively involved in what the attitude is about: first, because occupying the perspective of an attitude is an orientation in the space of reasons, and secondly, because such an orientation comes with normative pressure to take it at face value and manifest it in one’s patterns of action and reaction.

### Substantial mental attitudes

Spelling this out in case attitudes are substantial requires, first, a clarification of what it is to have a substantial mental attitude vis-à-vis X. What is it to consciously occupy the perspective of, say, caring about one’s job? It is to view one’s job as worthwhile, to regard it as important, i.e., to see it as significant to one’s life. More precisely, occupying the perspective of caring about one’s job is to see one’s job as a source of reasons. After all, deeming something important to oneself is to say that it provides one with reasons. If one cares about X, one is thus committed to a whole pattern of other mental attitudes and actions. For instance, if a person says that she cares about her job, she should also want to do her job well, regret missing an important meeting, make sure to put effort into her work and enjoy doing her job. Caring about her job commits her to the relevant kind of *engagement* on her part.^14^

This marks a fundamental difference between commitments inherent in trivial attitudes and in substantial attitudes. There seems to be a stronger sense in which a pattern of action and reaction isn’t only normatively implied but constitutively required for one to count as caring about something. If I don’t want to do my job well, nor put any effort into it or experience positive or negative emotions when things are going well or bad respectively, what does it even mean to say I care about my job? In such a case, I do not only appear to be ambivalent or irrational (or incoherent or inconsistent); my commitment to my job’s importance itself seems void. Where in the case of a non-substantial attitude, one can take something to be true while perhaps overlooking it again straight away, substantial attitudes seem to comprise commitments that do not allow sheer neglect. Hence, when attitudes are substantial, there is not only a normative but also *constitutive* connection between a substantial mental attitude and the relevant kind of engagement.^15^

One might wonder whether such a constitutive connection doesn’t bind us to a dispositionalist analysis. A dispositionalist would claim that having a belief *consists of* using it as a premise in reasoning or that it *causes* one to use it as such.^16^ Similarly, a dispositionalist would claim that caring about X *consists of* or *causes* the relevant patterns (cf. Naar 2013). In other words: the idea that the relevant patterns are constitutive of caring seems to be in line with such a dispositionalist analysis. Still, this need not bind us to a *strict* dispositionalist analysis. If commitment is seen as being on a par with other normative capacities such as reasoning, then there can be limits to the amount of failure allowed. If a person reasons invalidly, she fails to meet the requirements of reasoning validly, but not of reasoning altogether. But if a person moves from one thought to the other without any connection between them, she no longer counts as reasoning but rather as associating or letting thoughts flow by (cf. Kloosterboer 2022). Comparably, if a person commits herself to the importance of X, she can fail to fully integrate X in her life in the relevant ways. She then fails to completely fulfill her commitment without failing to *be* committed. But if she neglects X, does not regret her failure to integrate X in her life, is not answerable to any criticism, etc., then talk of commitment and failure becomes meaningless. We can thus see the constitutive connection between substantial attitude and engagement as a threshold of engagement that needs to be in place to make sense of the *strength* or *substantial* nature of the commitment inherent in substantial mental attitudes.^17^

Let me return to what this means for avowal and substantial self-knowledge. In the commitment view, to avow a substantial mental attitude regarding X is to express one’s commitment to X’s importance to oneself. This involves a commitment to taking X to be a source of reasons and thus comes with normative pressure to integrate X in one’s patterns of action and reaction. On this account, the relation between avowal and substantial mental attitude is thus *normative*: by avowing a substantial mental attitude, a person expresses her commitment to manifest it.

## The skeptical view

On this conception of avowal as involving a commitment, avowal’s importance in achieving self-knowledge is widely questioned. One of the most pressing concerns is that the link between avowal and self-knowledge only obtains when an attitude is ‘non-substantial’ or ‘trivial,’ not when it is ‘substantial,’ i.e., when it is significant to a person’s life and self-conception.^18^ As Schwitzgebel (2012, 193) writes:

If my attitudes – my beliefs and my values, especially – are not so much what I sincerely avow when the question is put to me explicitly but rather what is reflected in my overall patterns of action and reaction, in my implicit assumptions, my spontaneous inclinations, then although I may have pretty good knowledge of the simple and trivial, or the relatively narrow and concrete – what I think of April’s weather – the attitudes that are most morally central to my life, the ones crucial to my self-image, I tend to know only poorly…

The central point is that, given that substantial attitudes should be reflected not only in what I avow but also in my patterns of action and reaction, and given that a person’s avowal and patterns of action and reaction might diverge, she can only really know herself if she observes and interprets her actions and reactions (cf. Cassam 2014; Lawlor 2009; Schwitzgebel 2010, 2012). In this so-called skeptical view, avowal is neither a necessary nor essential condition of substantial self-knowledge.

Let me clarify the different tenets of the skeptical view. First, substantial mental attitudes are understood on said dispositional analysis. In the skeptical view, patterns of action and reaction constitute having the attitude. No normative connection between having the attitude and those patterns is presupposed in the analysis of substantial mental attitudes.

Secondly, what is needed to know such a disposition is not expressing a commitment (through avowal) but an assertion of having a disposition (the substantial mental attitude). Such an assertive self-ascription is true or false depending on whether the disposition is manifest in the relevant patterns of action and reaction. If a person self-ascribes a belief that *p* but this is not reflected in what she does, this shows that she does not have the belief, that her self-ascription was mistaken, and that she was thus ignorant about the belief.

Thirdly, as a result, the commitment expressed through avowal is seen as having few ramifications. In the skeptical view, an expression of a commitment to, for instance, the truth of *p* is just an expression of one’s occurrent thoughts on *p*. These occurrent thoughts may be different in a moment’s time or conflict with non-occurrent thoughts one has on *p*, and hence, are not taken to reveal anything significant about one’s mental life. In fact, in this view, avowals and occurrent thoughts might even be an obstacle in achieving self-knowledge, because they reflect the distorting lens of one’s self-conception – of how one wishes to appear to oneself.

Hence, in the skeptical view, to avow a substantial mental attitude regarding X involves expressing one’s occurrent thoughts on X. According to the skeptic, this is not only insufficient for but also has a distorting influence on acquiring substantial self-knowledge. Such knowledge consists of an assertive self-ascription instead. This is an assertion of having the relevant dispositions regarding X, which is true or false depending on whether the disposition is manifest in the relevant patterns of action and reaction. If a person really wants to know whether she has the substantial attitude, she should thus interpret her relevant patterns of action and reaction.

The expositions of the commitment view and the skeptical view show that their difference is driven by a different take on the nature of substantial mental attitudes.^19^ Hence, adjudicating between them depends not just on the question whether substantial self-knowledge involves avowal, but also on the nature of substantial mental attitudes. Can substantial mental attitudes, and substantial self-knowledge in its wake, be conceptualized without agency and the agent’s perspective? The arguments in the next two sections point out that they cannot: agency is at the heart of substantial self-knowledge.

## Self-knowledge without avowal

I will now turn to the first argument against the skeptical view, showing that denying any role for avowal in achieving substantial self-knowledge is untenable. I will argue against the idea that, as the skeptical view assumes, a substantial attitude can be *discovered* in patterns of action and reaction. To make the argument as strong as possible, this section adopts a crucial starting assumption of the skeptical view, to wit, that there is only a constitutive (and not a normative) connection between a substantial mental attitude and the relevant kind of engagement. The question pursued is whether and in what way this connection can be used epistemically, i.e., whether it can be used to “discover” the attitude in the engagement.

An influential example used to argue for the idea that one’s prior engagement is evidence for having an attitude is Krista Lawlor’s example of Katherine, who achieves self-knowledge by inferring whether she desires to have another child through internal promptings, such as that ‘she finds herself lingering over the memory of how a newborn feels in one’s arms’ and that ‘[s]he notes an emotion that could be envy when an acquaintance reveals her pregnancy’ (2009, 47). Internal promptings are imaginings, fantasies, memories, emotions and sensations and, according to Lawlor, ‘self-knowledge of desire is in routine cases a matter of self-interpretation of one’s imaginings, where that self-interpretation is a causal inference to the best explanation’ (2009, 62). But can internal promptings serve as evidence in the manner suggested by Lawlor? Does Katherine really need to (provisionally) *discover a fact about herself*, namely whether she does or does not want another child (cf. Lawlor 2009, 57)?

Lawlor’s case, as it is described, rejects the need for making an avowal: self-knowledge is acquired by paying close attention to one’s internal promptings and then inferring which mental attitude best explains these inner promptings. In responding to the case of Katherine, Boyle makes it clear that he doesn’t take such discovery to be a genuine possibility:

A person can certainly realize that she wants another child by paying attention to her own thoughts and feelings in the way Lawlor describes, but is it really plausible to represent this as a matter of detecting some standing fact of the matter? Her feelings when she boxes up outgrown clothes and receives news of her friend’s pregnancy are certainly indications of an incipient desire, but ‘incipient’ is important here. It is natural to imagine her also thinking of ways in which having another child would make it difficult to pursue other things she cares about. What she wants to know, presumably, is whether the decision to have another child is one she can genuinely embrace, and though ‘inner promptings’ may serve as indications of such a readiness, this is not simply a question of discovering what is already so but of reaching a settled attitude on the matter. To investigate this as if it were a matter for discovery on the basis of evidence sounds, even here, like alienation, or indeed like bad faith. (Boyle 2015, 344)

Boyle claims, first, that acquiring self-knowledge of the desire to have another child is not a matter of detecting a standing fact, but of reaching a settled attitude on the matter. Secondly, he claims that the reason for this is that such self-knowledge amounts to knowing whether the decision to have another child is one a person can genuinely embrace. Although I agree with the first claim, I think the second misrepresents the case. Boyle seems to offer a revision of the original case: on his portrayal of the case, Katherine needs to *decide* to have another child instead of merely determine whether she has a *desire* to have another child. Having a desire to have another child is not the same as viewing the desire as something to pursue *all things considered*. Katherine might have the desire to have another child, even if she thinks having another child would conflict with her desire to pursue her career and therefore, she cannot fully embrace the *decision* to have another child. After all, not all things that one deems to be good can be pursued at the same time.^20^ Referring to decision (and its commitments) thus doesn’t solve the question raised by Lawlor’s example. Namely: why is it problematic to claim that internal promptings are *evidence* for having the desire? Can’t we imagine that Katherine would experience internal promptings to such a degree that she cannot but infer that she has the desire?

If internal promptings are to be evidence, however, two questions need to be answered. First, how does Katherine know it is envy that she feels? In the description of the case, Lawlor writes that Katherine ‘notes an emotion *that could be envy* when an acquaintance reveals her pregnancy’ (2009, 47. Italics mine). The problem reflected in this passage is that, like desire itself, internal promptings are often *mental attitudes* one needs to know about. Before Katherine can take her envy to be indicative of her desire to have another child, she first needs to recognize her emotional reaction *as envy*. How does such recognition take place? How does Katherine know that the emotion she feels is envy?

One possible answer is to say that Katherine knows this because of yet other internal promptings that could be taken as evidence for it. Such a response isn’t possible *ad infinitum*. On pain of infinite regress, it must be possible to know some internal promptings by following a different path. In response, Lawlor might argue that, indeed, many internal promptings can be known differently; only substantial mental attitudes are inferred from internal promptings. Or one might claim that self-knowledge is holistic: self-interpretation of X presupposes other self-knowledge (cf. Cassam 2014, 169). This makes self-knowledge circular, but not necessarily viciously so. I wonder whether these different paths to knowing internal promptings do not involve avowal. But asking this question only reiterates the assumptions of the commitment view, which would not convince those advocating the skeptical view. Fortunately, I think that the problem with Lawlor’s account can be explained independently.

Suppose that internal promptings are known in a different way, not involving avowal; how do internal promptings acquire their evidential significance? How does Katherine *know* what her internal promptings *indicate*? How does she know how to delineate the pattern that they are part of? How does Katherine know that, e.g., her envy reveals a deeper truth about herself and isn’t due to, for example, just having a grumpy day? The envy – the internal prompting – does not itself tell us whether it is a symptom of a deeper desire. After all, emotional episodes have different kinds of significance for a person. Among other things, a person may discard them for making a fuss about something insignificant, or she may experience them as an *expression* of what she cares about. It therefore seems that Katherine’s envy cannot be seen as *plain evidence* for her deeper desire.

This idea is reflected in an argument made by Richard Moran. As Moran writes, it is impossible to treat one’s entire mental life as mere data: ‘a person cannot treat his mental goings-on as just so much data or evidence about his state of mind all the way down’ (Moran 2001, 150). Even if, in principle, it is possible to treat any mental attitude as a mere datum, it is impossible to treat my entire mental life as mere data: in each case of treating a mental attitude as datum, I must also, at some point, orient myself in the space of reasons.^21^ Delineating patterns of action and reaction, determining what is central to a pattern, which attitudes to consider, why envy is in one case significant or insignificant, whether the envy is anomalous or gives meaning to a pattern, all these issues require an agent, who not merely interprets herself from a detached perspective, but actually thinks about what goes on in her life. Independent of the *method* of knowing an internal prompting, determining its significance depends on the perspective of the agent – on occupying the perspective of at least some of the attitudes in the pattern. Hence, Katherine’s internal promptings are evidence for having the desire to have another child only if they are related in the relevant way to ‘internal promptings’ the perspective of which she consciously occupies and which she can thus avow.^22^

The upshot of this is that it is misguided to portray the subject’s relation to her ‘internal promptings’ (or her *engagement* with the object of her substantial attitude) as merely passive. Internal promptings aren’t just *given facts* about the subject that she can discover, experience, or note, but are (at least some of them are) an expression of the subject’s commitments to what goes on in her life – an expression of her orientation in the space of reasons. As I have hoped to show, this means that a subject’s prior actions and reactions cannot be taken as plain evidence, sufficient to determine whether she has a specific mental attitude. Avowing a mental attitude should accord with one’s patterns of action and reaction, but that does not mean that these patterns can *replace* avowals completely.^23^ The skeptical view is thus mistaken in thinking that substantial mental attitudes can be *discovered* in the relevant engagement, because *knowing* which engagement is relevant and what it signifies depends on avowal.

## Substantial attitudes and failing to live up to them

The second argument against the skeptical view concerns the significance of a gap between avowal and engagement. The skeptical view claims that the possible and prevalent gap between avowal and engagement shows that avowal is an unreliable guide at best. This way of thinking, as I will argue in this section, neglects an intuitive difference between failing to know one’s substantial mental attitudes and failing to live up to them. If we want to hold on to this distinction, then we need avowal that expresses commitment.

It seems abundantly clear that it is possible and prevalent that we fail to live up to our avowals. I say I believe that taking care of the environment is important but fail to actually take care of the environment (or at least choose the alternatives that do less damage to the environment). I say I care about my health but fail to establish healthy habits. I say I care about my job but find myself struggling to see the value of what I do. Following such examples, the skeptical view can easily argue that my avowal of a substantial attitude cannot be an expression of a commitment *that I already have*, but rather is something more *provisional*, like trying to be committed in that way. Whether I have the commitment then is a question that can only be answered either by checking with my future engagement itself or by knowing how likely it will be that I will be engaged in the appropriate way (e.g., I have done so in the past; I have good self-regulation skills).^24^ In this picture, an avowal of a substantial attitude must be corroborated in (the likelihood of) the relevant kind of future engagement if it is to amount to knowing the substantial attitude. Such corroboration is needed because of the possibility and prevalence of a gap between avowal and engagement.^25^

Based on the assumptions of the skeptical view this is obviously true. If avowal is seen as a statement of having a disposition and engagement as the manifestation of that disposition, a gap between avowal and engagement implies ignorance or unreliability. However, in the commitment view, such a gap need not necessarily imply ignorance. If the relation between avowal and engagement is (also) normative, then by avowing a substantial mental attitude a person is not merely stating how things will likely turn out to be. Instead, she is committing herself to live her life in a certain way. In avowing my care for a particular friend, I am not just conveying my certainty of my future behavior regarding her; I am expressing my commitment to stay true to our friendship.

The normativity of commitment (and of our existence) is a theme widely explored in continental philosophy, especially the existentialist tradition^26^, but also in the analytic tradition.^27^ One important aspect of commitment being normative is the idea that you can fail to live up to it. After all, normativity is prescriptive: it lays down requirements one should fulfill. Consider a norm of valid reasoning. Precisely because it *prescribes* how one should reason – and doesn’t *describe* how human beings necessarily do reason – it is something one can fail to live up to. Inherent to normativity is the idea of failure: one can fail to manifest what is prescribed. Something similar can be said of commitment. If an agent commits herself to something, it is indeed not yet manifest, which implies she can also fail to manifest it. Portraying the relation between avowal and the relevant engagement as normative thus has consequences for what a gap between avowal and engagement might indicate. I will explicate these consequences in the rest of this section. Since the argument proceeds from the assumption that avowal’s relation to engagement is in part normative, it might not convince those advocating the skeptical view. As I will show, however, that comes at a cost.

### The significance of a gap between avowal and engagement

The first question to ask is how to infer from the mere appearance of a gap between a person’s avowals and her patterns of action and reaction what such a gap implies. How should it be determined what a possible or real gap between avowal and (future) engagement indicates? The problem is that observing a person’s engagement does not determine 1) whether the gap between avowal of one’s substantial attitude and future engagement actually constitutes a *failure* to live up to one’s avowed care, nor 2) whether that failure constitutes a *failure to know* one’s substantial attitude. First, it cannot be concluded that a person fails to live up to her avowed attitude by observing her engagement because a gap between avowal and engagement might also be the result of a change of mind (or heart). For instance, I could believe that *p* and know this of myself, and after a change of mind (due to, for instance, learning new information about the issue) believe that *q* instead of *p* and still know this of myself. Similarly, I could care about X and, after enough time, have a change of mind (or heart) and stop caring about X. This need not impugn that I knew I cared about X.^28^ Whether a gap between avowal and (future) engagement is a failure or a result of a change of mind is not something that can be observed in the engagement itself. This reflects the situation of knowledge of one’s actions: if I get up to make green tea and, while I am making tea, make lapsang souchong instead, you must ask me whether I made a mistake or whether I changed my mind (cf. Falvey 2000).

Secondly, one can fail in (fully) manifesting a substantial mental attitude. We are human, after all. We sometimes let down our loved ones, struggle with making fully sustainable choices, or we fail to prioritize our occupations in line with our values. But such failures are only conceivable if one *has* the substantial mental attitude: failing to live up to an attitude presupposes having it. Hence, if we think such failures are possible, which seems the only plausible option to me, then a distinction between failing to live up to and failing to know one’s attitude must be presupposed. This implies that it cannot be concluded from failing to live up to one’s avowed attitude that one fails to know it. Thus, the fact of a gap between avowal and engagement does not necessarily imply ignorance.

As mentioned in section 2, there are limits to the amount of failure if one is to count as having the substantial attitude. For instance, failing on too many or too important occasions undermines caring about something, or at least reveals an ambivalence in the person’s attitudes. But like the difference between a change of mind and a failure, the difference between failing to live up to and failing to know one’s attitude isn’t *observable* in the engagement itself. There isn’t a clear division between a failure to live up to one’s care about X and a failure to care about X (and thus a failure to know it). Rather, what counts as a certain kind of failure can be viewed as the result of a complicated process in which both the person herself reconsiders whether she cares about X (and thus whether X is important for her) and negotiates with others whether her engagement with X, including failures to have the relevant kind of engagement, suffices for caring about X.^29^

Schwitzgebel (2012) seems to neglect the difference between failing to live up to and failing to have a substantial mental attitude. He gives the following example:

I say I value family over work. When I stop to consider it, it seems to me vastly more important to be a good father than to craft a few more essays like this one. Yet I’m off to work early, I come home late. I take family vacations and my mind is wandering in the philosopher’s ether. I’m more elated by my rising prestige than by my son’s successes in school. My wife rightly scolds me: Do I really believe that family is more important?

For Schwitzgebel, this example demonstrates our self-ignorance: we say (or avow) one thing but behave to the contrary. Schwitzgebel’s argumentation clearly is exemplary of the skeptical view. Suppose we agree with Schwitzgebel that the things he describes constitute failures to live up to valuing family over work, and thus agree that his wife ‘*rightly’* scolds him, do we then *know* whether Schwitzgebel values family over work or vice versa? Drawing such a conclusion would assume, first, that these things can be taken as *plain evidence* for his values. As argued in section 4, such evidence is not sufficient to determine which of the two Schwitzgebel values more. Secondly, such a conclusion assumes that his mistakes are unambiguous signs of his self-ignorance. But it seems just as plausible that his failures do not indicate self-ignorance, but that he just fails to live up to his values. Which of the two it is will be a matter for discussion. Not for us as philosophers, but for Schwitzgebel and his wife: he needs to reconsider, and negotiate with his wife, whether he values family over work and, importantly, what kind of engagement regarding his family is actually demanded by the care he has for his family. For instance, is it really problematic that, even during family vacations, his mind is wandering in the ‘philosopher’s ether’? Or that he spends a lot of time writing essays? Answers to these kinds of questions are, I think, not clear-cut but a matter of negotiation both with oneself and with others. Hence, a perceived gap between avowal and engagement is just that: a perceived gap. Its significance depends on the person’s own perspective and thus on avowal.

### Is there a standard of engagement?

There is even more reason to be wary of the simple picture of the relation between avowal and engagement sketched in the skeptical view. Since the skeptical picture assumes the possibility of checking whether a person is engaged appropriately (in the future), it must presuppose that there is some set standard regarding how she should be engaged. But is there really such a standard?

Consider Robert Pippin’s analysis of what is involved in knowing one’s practical identity. In an essay on Proust and self-knowledge, Pippin (2005, 315) describes Marcel’s (search for) knowledge of being a writer in the following way:

The young Marcel considers himself, from very early on, a writer; that is his self-understanding; and he is very much trying to become who he believes he is, trying to become a writer. And this is indeed portrayed as a struggle… For a very long time… Marcel is a writer who does not write or writes very little as he struggles to understand how a writer lives, how one responds to and tries to understand the people around him “as a writer would” and struggles to find out whether he can ever become in reality, however much he actually writes, “a real writer.”

Pippin portrays Marcel’s struggle to become who he considers himself to be – to make his self-conception true – not only as a struggle to actually write but also as a struggle to understand what it means to be a writer. Especially, it is a struggle to understand, amidst Marcel’s own and society’s expectations of how a writer should live, what it means for *himself* to be a writer. Such an understanding, as argued by Pippin, cannot be achieved by mere theoretical means – not by contemplating the life of a writer nor by searching for one’s own “writerly essence” (2005, 331). It is instead a matter of trying to *be* a writer: in the act of writing, in failing to write, and in negotiating with others what it is that one is doing, one can start to understand what it means for oneself to be (or failing to be) a writer.

In a similar vein, the relevant kind of engagement belonging to a substantial attitude, especially what that is *for a particular person*, does not seem to be clear-cut either. Nor does it seem possible that understanding the relevant kind of engagement can be the result of theorizing about what it means to have that attitude. Rather, such understanding seems to depend on trying to live up to the attitude. In the case of caring, for instance, knowing what it means for a person to care about X, and thus what kind of engagement is involved, seems to be something only she can understand, and only by actually trying to care about X. I will attempt to explain this more precisely.

To see the lack of a determined standard of engagement in the case of caring, and thus the necessity to actually *engage* in order to understand this standard, consider the following example. Suppose Joan avows that she wants to spend the rest of her life with David^30^ (for brevity’s sake, let’s say she wants to be *married* to David). And suppose we say that she should assess the likelihood of her staying faithful to David and of her being able to spend her whole life with him, in good and bad times, so as to know whether she *really* wants to be married to him. On what would she base such an assessment? Her commitment to David in the past? Her resilience in dealing with temptations and setbacks? Obviously, these sorts of things matter. Joan’s avowal that she wants to be married to David must accord with the patterns of her actions and reactions. But do these sorts of things provide information about what it means to spend the rest of her life with David? What it means to grow old together? What it means to take care of each other for the rest of their lives? Joan will have ideas about these things, she will have imagined her future with David repetitively, and she will harbor expectations about how her life with David will be. In other words, her wanting to be married to David is tied to a conception of how her life with David will be, and to a self-conception of the person she will become having David as her husband and being his wife.

But in trying to be that person and in trying to have the life she imagined, she will unquestionably experience tension between her expectations and how it ultimately turns out. She might doubt whether she can endure the fights they have and, next, doubt whether her doubt can be part of wanting to be married to David. She might experience difficulties in accepting David’s growing fondness for taking long, solitary walks. This presents her with the question of how many experienced difficulties in marriage will be acceptable *for her* and with the question of when *she* will stop wanting to be married to David. Such questions aren’t answered by theoretical reflection but by experiencing doubt and by returning to one’s sense of commitment (or failing to do so), or by experiencing the difficulties and finding a way (or failing to find one) to accept or deal with them. Such experiences and challenges put into question whether Joan sees herself as the kind of person who is committed to being married to David under the current circumstances. Hence, in both these ways – i.e., in understanding which commitments are inherent in wanting to be married to David and in truly understanding the commitment itself – grasping the meaning of wanting to be married to David can only come about by trying to live the rest of her life with him.

One might think that the tension Joan experiences is reason for her to detach herself from her agential, engaged perspective and to reflect on her patterns of action and reaction from a more objective point of view instead. Could that help her in determining whether she wants to be married to David? Suppose she considers a whole range of actions, affections and cognitions regarding her marriage, i.e., her anger, doubt, sadness and presumably also her joy, deep affection, taking care of each other, feeling safe and at home. What is the next step that Joan should take from this detached viewpoint? Figure out what the pattern adds up to? How would that even work? Perhaps it is clear to Joan that the pattern does not add up to a preconceived romantic ideal of marriage. But then what? Why should she hold on to that conception? Or perhaps, during a fit of anger, she loathes David and cannot imagine sleeping in the same bed at night. Still, she also knows that she gets so angry with him because she cares so much for him. She knows that fits of anger come and go. She knows that her patterns of action and reaction contain ambiguous elements. Should such patterns be coherent? Isn’t ambiguity part of the human condition? What else can Joan bring into her reflections to reach a conclusion? I really do not know.

This short exercise of trying out a detached perspective shows that even if Joan’s reflections might take such a form, she cannot reach a conclusion without taking a stance: on what marriage should be like, on how much ambiguity is allowed, on the human condition, and, more specifically, on what all these things mean to *herself*. Wanting to be married to someone is not something that can be calculated; rather, it is an exercise in living, in coming to understand who one is and what forms of marriage one can be committed to.

Let’s take stock. I have argued that the skeptical view where one’s future engagement plays the role of evidence in knowing whether one has a substantial mental attitude is mistaken, first, because one cannot observe whether a gap between an avowed substantial attitude and the relevant engagement is due to a failure to have the attitude. And secondly, because the required engagement isn’t set in stone, but rather is something the person herself must come to understand in trying to live up to the commitments inherent in the attitude. Should those adhering to the skeptical view accept this conclusion? Not necessarily, but if they reject it, they will have to deny the plausible distinction between failing to live up to and failing to have a substantial attitude. Additionally, they will have to show how the relevant kind of engagement can be determined without the agent’s own perspective.

## Concluding remarks

In this paper, after clarifying the commitment view and the skeptical view on the status of avowal in substantial self-knowledge and explicating their underlying differences, I have presented two arguments in favor of the former. I have first argued that a constitutive relation between having a substantial mental attitude and manifesting the relevant patterns of action and reaction cannot be used evidentially without avowal. Patterns of action and reaction aren’t just *given facts* about a person that she can discover, experience, or note. They only have significance as evidence if, at some point, the person does orient herself in the space of reasons.

The second argument focused on how the existence and significance of a gap between avowal and engagement is to be determined. First, heeding the distinction between failing to live up to an avowed substantial attitude and failing to have it, means that the *significance* of a gap between avowal and engagement cannot be determined without the agent’s own (avowed) perspective. Secondly, due to a lack of predetermined and universal standard of engagement, it is even difficult to determine the actual *existence* of a gap without the agent’s own perspective. If certain tensions between avowed attitude and engagement are revealed, it is up to the person herself (in relation to and in negotiation with others) to determine whether she fails to live up to her attitude or fails to have it, what engagement is required by living up to the attitude, and whether she can return to her sense of commitment. The skeptical view could reject this conclusion, but only at a significant cost: they would have to deny the plausible distinction between failing to live up to and failing to have a substantial attitude and they would have to show how the relevant kind of engagement can be determined without the agent’s own perspective.

Let me finish this paper with a sketch of the crucial role of avowal in achieving substantial self-knowledge. What I have argued is that the engagement involved in having a substantial mental attitude requires trying to live up to it. But one can only try to live up to something if one is committed to the thing in question. How does an agent know whether she is committed to something? Indeed, she knows this through *avowal*, in which one expresses one’s commitment. Importantly, this is not meant to say that one cannot care about X without avowal. The claim concerns the possibility of *knowing* one cares about X and, in proximity, of making caring about X one’s business, of taking responsibility for the persons, ideals and things one cares about.

None of this is to say that avowal is *sufficient* for substantial self-knowledge, just that avowal is necessary. The point is that without avowal, achieving self-knowledge of one’s substantial attitudes cannot even begin. Avowing one’s care, and thus being committed, may only provide knowledge that is *provisional*. However, this is not due to the inferior status of avowal but to the nature of substantial attitudes: after all, they involve commitments that must be fulfilled, and as such they extend into the future. Moreover, what this precisely means for a particular person in particular future situations, is not ready-made. Hence, to see whether a person is engaged appropriately, she herself must be in the business of committing herself and finding out about the relevant engagement. In this alternative picture, substantial self-knowledge does not put us back in the observer’s seat but makes the agential aspects of self-knowledge even more pertinent. The idea that having a substantial mental attitude is something one needs to live up to, and especially, that one can fail to live up to, implies that it is an attitude only *agents* can have. It thus seems that avowal is *par excellence* essential to substantial self-knowledge.^31^

## Footnotes

1 I follow the literature in characterizing avowal this way. See, especially, Moran (2001). Although I will not engage in Moran-exegesis, nor defend Moran’s account of self-knowledge, the thoughts developed in this paper are greatly influenced by Moran’s writings. For more on the role of commitment and avowal in self-knowledge, see, e.g., Bilgrami (2006), Boyle (2011a), Coliva (2016), Finkelstein (2003), Moran (2001) and Kloosterboer (2015). For a different take on avowals, e.g., in expressivist accounts of self-knowledge, see Bar-On (2004).

2 What this commitment precisely amounts to, and how it relates to the rationality of the attitude and one’s epistemic evaluation of the attitude, will be explored in section 2.

3 Cf. Cassam (2014); Lawlor (2009); Schwitzgebel (2010, 2012).

4 These responses in the literature defend Moran’s account of self-knowledge. See, especially Boyle (2015). Other responses with a similar strategy, i.e., accepting that Moran applies only to non-substantial self-knowledge, can be found in Schwenkler (2018), and Gertler (2016). What is quite striking in this respect is that such acceptance is, as far as I can tell, absent in Moran’s view. He doesn’t talk of non-substantial (aka ‘trivial’) or substantial self-knowledge, but he does claim that his account applies not only to beliefs but also to emotions and intentions (Moran 2001, 64-5; 2012, 214; 2004, 471). Moreover, the examples that Moran turns to are often examples of substantial self-knowledge: for instance, the case of the analysand (2001, 93-5); akratic gambler (2001, 78-82; 162-3); the rakehell (2001, 174-187); and Fred Vincy (2001, 188-192).

5 See McGeer (1996; 2007). McGeer’s position follows the skeptic in accepting a conflict between the claim that avowal is essential to self-knowledge and the claim that our words and deeds often don’t match. However, she thinks that the solution is to bring our words and deeds into better alignment through self-regulation skills. Thanks to an anonymous reviewer for asking to clarify the relation between McGeer’s view and the skeptical view.

6 Importantly, this claim holds for self-knowledge of *intentional mental attitudes*. These attitudes, such as beliefs, emotions, desires and intentions, are fundamentally different from sensations, headaches and heart rates because they involve, for the subject of those states, a characteristic grasp of the world. That is to say that these attitudes involve, from a first-person perspective, grasping the (propositional) object of those states *as true*, *as to be done*, *as dangerous*, etcetera. See, especially Bilgrami (2006); Moran (2001).

7 This is one commitment that is being expressed in fear. One other crucial commitment being expressed is that the communal garden is something the agent cares about. See Kloosterboer (2015).

8 In much contemporary analytic philosophy, reflective endorsement is often considered as a form of stepping back from one’s attitude and then, based on evidential considerations, judging it to be true, rational, or epistemically justified. See, for instance, how Leite (2018) presents the view that believing or attributing a belief involves its endorsement (to then provide objections to it). For a different view of what kind of mental agency the notion of “reflective endorsement” is supposed to capture, see especially Boyle (2009; 2011b). Because I want to focus on substantial self-knowledge, I do not have space to delve into the debate on reflective endorsement. Instead, I stick to the notion of commitment.

9 Doyle (2019, 443ff) explicates the idea of consciously occupying the perspective of an attitude in terms of phenomenology, e.g., feeling compelled. I think this can also be seen as an orientation in the space of reasons, if that space of reasons is understood in broader terms than reasons that we reflectively endorse (in its dominant understanding). For reasons explored in the main text, I think we need this idea of orientation in the space of reasons to make sense of the normativity of commitment.

10 For more on this connection, see especially the literature on mental agency, transparent self-knowledge and Moore’s paradox. For instance, see Bilgrami (2006), Boyle (2009; 2011b), Hieronymi (2009; 2013), Larmore (2010), Moran (2001), Van Woudenberg & Kloosterboer (2019).

11 Compare Anscombe (1957) and Moran (2001).

12 Rik Peels suggested to me that one could say that the normative pressure is *defeasible*. However, I think the normative pressure does not disappear, but may turn the other way, i.e., towards revising the belief.

13 What might come up in my practical reasoning is not the perspective of fear but the fact that I fear flying – a psychological fact about me. In my practical reasoning, I distance myself from the perspective of the fear. Cf. Anscombe (1989). Sometimes conflicting emotions attach to reasons I *should* consider and hence show I should revisit my all-things-considered judgment. Cf. Jones (2004).

14 Cf. Arpaly (2003); Helm (2010); Smith (2005); Seidman (2016). In Helm’s terminology, caring about X means that X is the focus of a pattern of emotions, desires, judgments, intentions and actions (2010, 311-5). According to Arpaly, caring is constituted by three types of engagement: a motivational, emotional and cognitive one (2003, 85-7).

15 As will become apparent further on, this is not to say that one cannot fail to live up to commitments inherent in caring about something. Like belief (as will be discussed further on), such failures seem to be possible, and one can be subject to rational criticism for not fulfilling one’s commitments or subject to questions about having those commitments in the first place. But to reiterate the point, however ambivalent or complex caring about something or someone might be, a minimal kind of engagement with what one cares about needs to be in place.

16 See, for example, Audi (1972; 1994), Price (1969), Schwitzgebel (2002; 2013). My use of *dispositionalism* in this paper is limited to refer to these accounts, not to accounts that analyze capacities or abilities in terms of dispositions. See, for example, Vetter (2019).

17 Much of this is in line with Akeel Bilgrami’s notion of commitment. One might think that the constitutive connection between commitment and engagement when attitudes are substantial conflicts with Bilgrami’s claim that one can have commitments, and avow these truly, even when one is 'not supposed to do anything' to live up to them (2006, 227). However, Bilgrami (2010, 756) also writes that ‘[a]ll we need to find in order to attribute the commitment to him, is that when and if he does become aware of his dispositions and notices his failures to live up to his commitments, he accepts criticism for not living up to his commitments, and tries to do better by way of living up to them…' On my account, this is a manifestation of *a* relevant (but partial) pattern of actions and reactions. This was brought to my attention by an anonymous reviewer.

18 Although “trivial” and “substantial” might not be the best terms to depict these different kinds of self-knowledge – after all, what is termed “trivial” in this distinction is *also* crucial in our lives – I stick to this terminology for simplicity’s sake.

19 Thanks to an anonymous reviewer for asking me to reflect on this.

20 Hence, this isn’t meant to disqualify the thesis that desire operates ‘under the guise of the good.’ See Boyle and Lavin (2010). Desire is an orientation in the space of reasons (i.e., towards the good), but not necessarily an expression of an all-things-considered judgment.

21 As stated by Moran, ‘[a]t some point, I must cease attempting to infer from some occurrence to my belief; and instead *stake* myself, and relate to my mental life not as something of symptomatic value, but as my current commitment to how things are out there’ (2001, 150). See also Moran (2001, 121-124).

22 Wouldn’t Lawlor simply reject that Katherine needs to *avow* her internal promptings and instead claim that they might just *feel* they are expressive of her perspective? Can’t she just turn to phenomenology? In response, I take it that an internal prompting can only feel expressive of one’s perspective if it is an expression of one’s orientation in the space of reasons. This is precisely what avowal is about. Similarly, Cassam’s holism seems to depend on a subject that can also consciously occupy the perspective of (many of) her mental attitudes, not least of all the perspective of her interpretive reasoning and conclusion. The perspective is not a detached perspective all the way down. This will become even more clear in section 5.

23 As Moran (2001, 163) writes: ‘The assertion from the Deliberative stance that “I am not bound by my empirical history” is not in any way a denial that the facts of my history are what they are. It does not deny either the truth of these claims or their relevance to the question at hand; but it does deny their completeness and, in a word, their decisiveness.’ See also Moran (2003, 20). Furthermore, see, for instance, *Anscombe’s principle* as formulated by Setiya (2011, 174): ‘If A has the capacity to act for reasons, she has the capacity to know what she is doing without observation or inference – in that her knowledge does not rest on *sufficient prior evidence*’ (italics mine). See Falvey (2000) for an insightful discussion of the implications of such a claim in the case of intention and action.

24 This is in line with McGeer’s (2007) approach.

25 This reflects the argument from illusion, which assumes, roughly, that the possibility of being wrong (being under the sway of an illusion) implies the need for external justification. Cf. Dancy (1995).

26 See, especially, Sartre (1956), but also Larmore (2010); Moran (2001); Taylor (1976; 1989).

27 See, for instance, Anscombe (1957); Bilgrami (2006); Boyle (2009, 2011a, b); Geach (1957); Hampshire (1975); Hieronymi (2009); Korsgaard (1996, 2009); Wittgenstein (2009).

28 This is not to say that any change of mind (or heart) is acceptable. If the period is too short, if the change of mind happens randomly, if I change my mind (or heart) repeatedly or very often, it becomes doubtful whether my care for X is genuine. In situations like these, having a change of mind (or heart) does seem to constitute a failure to live up to one’s avowal. See also main text.

29 This latter condition, i.e., negotiating with others, is part of the social dimension of self-knowledge, which I cannot do full justice to in this paper due to complexity and lack of space. See McGeer (2007) for the social dimension of developing the requisite capacities for first-person authority. And see Pippin (2005, 309, 318-322) for a discussion of the influence of negotiation with others.

30 David could, of course, also be another woman. Being of different gender, however, makes it easier for writing and reading comprehension, because one can more easily differentiate by referring to “he”, “she”, “him” and “her”.

31 Many thanks to Lieke Asma, Leon de Bruin, Gerrit Glas, Fleur Jongepier, Niels van Miltenburg, Rik Peels, Beate Roessler, Johannes Roessler, and René van Woudenberg for their encouragement and comments on earlier versions of the paper. I would also like to thank the students participating in the Core Seminar co-taught with Niels van Miltenburg at Utrecht University in 2020 and Sally Farrar for her support in finding my own voice in English.
